# Short-Term Treatment with Bisphenol-A Leads to Metabolic Abnormalities in Adult Male Mice

**DOI:** 10.1371/journal.pone.0033814

**Published:** 2012-03-28

**Authors:** Thiago M. Batista, Paloma Alonso-Magdalena, Elaine Vieira, Maria Esmeria C. Amaral, Christopher R. Cederroth, Serge Nef, Ivan Quesada, Everardo M. Carneiro, Angel Nadal

**Affiliations:** 1 Departamento de Anatomia, Biologia Celular, Fisiologia e Biofísica, Instituto de Biologia, Universidade Estadual de Campinas, UNICAMP, Campinas, Sao Paulo, Brazil; 2 Instituto Nacional de Ciência e Tecnologia de Obesidade e Diabetes, Sao Paulo, Brazil; 3 Instituto de Bioingeniería, Universidad Miguel Hernández de Elche, Elche, Spain; 4 Centro de Investigación Biomédica en Red de Diabetes y Enfermedades Metabólicas Asociadas, CIBERDEM, Elche, Spain; 5 Department of Genetic Medicine and Development, University of Geneva Medical School, Geneva, Switzerland; 6 Centro Universitário Hermínio Ometto, Programa de Pós Graduação em Ciências Biomédicas - UNIARARAS, Araras, Sao Paulo, Brazil; Pennington Biomedical Research Center, United States of America

## Abstract

Bisphenol-A (BPA) is one of the most widespread endocrine disrupting chemicals (EDC) used as the base compound in the manufacture of polycarbonate plastics. Although evidence points to consider exposure to BPA as a risk factor for insulin resistance, its actions on whole body metabolism and on insulin-sensitive tissues are still unclear. The aim of the present work was to study the effects of low doses of BPA in insulin-sensitive peripheral tissues and whole body metabolism in adult mice. Adult mice were treated with subcutaneous injection of 100 µg/kg BPA or vehicle for 8 days. Whole body energy homeostasis was assessed with *in vivo* indirect calorimetry. Insulin signaling assays were conducted by western blot analysis. Mice treated with BPA were insulin resistant and had increased glucose-stimulated insulin release. BPA-treated mice had decreased food intake, lower body temperature and locomotor activity compared to control. In skeletal muscle, insulin-stimulated tyrosine phosphorylation of the insulin receptor β subunit was impaired in BPA-treated mice. This impairment was associated with a reduced insulin-stimulated Akt phosphorylation in the Thr^308^ residue. Both skeletal muscle and liver displayed an upregulation of IRS-1 protein by BPA. The mitogen-activated protein kinase (MAPK) signaling pathway was also impaired in the skeletal muscle from BPA-treated mice. In the liver, BPA effects were of lesser intensity with decreased insulin-stimulated tyrosine phosphorylation of the insulin receptor β subunit.

In conclusion, short-term treatment with low doses of BPA slows down whole body energy metabolism and disrupts insulin signaling in peripheral tissues. Thus, our findings support the notion that BPA can be considered a risk factor for the development of type 2 diabetes.

## Introduction

The incidence of Type 2 diabetes mellitus is growing and millions of people are diagnosed with this metabolic disorder every year. The characteristic features of this disease include glucose intolerance, insulin resistance in skeletal muscle, adipocytes and liver, and often compensating hyperinsulinemia at the onset of this pathology. At later stages, β-cell function is affected leading to a decrease in insulin release and lower levels of plasma insulin, which favors hyperglycemia. Insulin resistance can develop in response to the environment and results from a complex interplay between nutrient overload, systemic excess of fatty acids, adipose tissue inflammation and oxidative stress [1]. In recent years, the life style in the modern society has drastically changed, with increased consumption of fat-rich food and sedentary activities among other factors. Thus, nowadays, an excessive caloric intake and an inadequate physical activity have become the most important players in the development of insulin resistance [2]. In addition, exposure to endocrine disrupting chemicals (EDCs) resulting from life style changes, such as consumption of canned food or drinks, as well as food embedded in plastics, was also proposed to be involved in the etiology of insulin resistance and associated metabolic disorders [3]–[5]. Recently, considerable attention has been given to the endocrine disruptor bisphenol-A (BPA), since epidemiological studies in humans have associated bisphenol-A exposure with an increased risk of adverse health effects including diabetes and insulin resistance [6]–[8]. BPA, which possesses xenoestrogenic activity [9], is currently used as the base compound in the manufacture of polycarbonate plastic and the resin lining of food beverage cans as well as in drinking water bottles and stores [10]. A large number of *in vivo* and *in vitro* studies have reported adverse effects of BPA and a significant number of these were performed below the predicted “safe” reference dose of 50 µg/kg/day, established by the U.S.-EPA [6]. Importantly, this safe exposure level is 1,000 times lower than the amount found to produce the lowest adverse effect for BPA in laboratory animals established by the lowest-observed-adverse-effect-level (LOAEL) (50 mg/kg per day).

Recently, pharmacokinetic experiments performed in monkeys indicated that BPA exposure may be much higher than initially thought [11] and exposure may originate from sources other than food, i.e, skin absorption [12], [13]. BPA exposure has already been demonstrated to be widespread as it was found in more than 93% of USA citizens [14] and its concentration in blood reaches 1–18 nM [6].

Studies in rodents have demonstrated that exposure to BPA as well as other EDCs elicits alterations in glucose homeostasis [15]–[18]. We have previously shown that BPA has a direct effect on pancreatic β-cells potentiating glucose-stimulated insulin secretion, which favors postprandial hyperinsulinemia as well as insulin resistance [9], [19]. Remarkably, insulin resistance appeared when mice were exposed to BPA orally or by subcutaneous injection [15]. In addition to a direct modulation of pancreatic β-cell function, BPA exposure also involves actions on peripheral metabolic tissues [15], [16]. In the present study, we confirm our previous results regarding the potential diabetogenic effect of BPA on glucose homeostasis and we further demonstrate that eight days treatment with low doses of BPA alters whole body energy homeostasis and impairs insulin action in skeletal muscle and liver. Thus, the present results indicate that BPA disrupts insulin signaling in peripheral tissues and is likely a risk factor for the development of type 2 diabetes.

## Materials and Methods

### Animals and treatment

The ethical committee of Miguel Hernandez University “Comisión de Ética en la Investigación Experimental” specifically reviewed and approved this study (approval ID: INA-AN-001-07). Experiments were performed with 3 months old male Swiss albino OF1 mice that were individually maintained under standard housing conditions. BPA was dissolved in tocopherol-stripped corn oil and administered subcutaneously during 8 days twice a day (9:00 a.m. and 2:00 p.m.). Control mice were injected with 100 µl of vehicle at the same time-points. The total daily dose used for BPA was 100 µg/kg.

### Plasma analysis

To measure plasma metabolites blood samples were collected from the tail vein. Insulin was measured by enzyme-linked immunosorbent assay (Mercodia, Crystal Chem, Sweden) and NEFA was measured using a commercial kit (Wako®; Richmond, USA).

### Glucose and insulin tolerance test

For glucose tolerance tests, animals were fasted overnight for 12 hr, and blood samples were obtained from the tail vein. Animals were then injected intraperitoneally with 2 g/kg body weight of glucose, and blood samples were taken at the indicated intervals. For insulin tolerance tests, fed animals were used. Animals were injected intra-peritoneally with 0.75 IU/kg body weight of soluble insulin. Blood glucose was measured in each sample using an Accu-check compact glucometer (Roche, Madrid, Spain).

### Insulin secretion measurement

Pancreatic islets of Langerhans were isolated by collagenase digestion of the pancreas. For static incubations, four islets from each experimental group were first incubated for 30 min at 37°C in Krebs–bicarbonate (KBR) buffer with the following composition: NaCl 115 mmol/L, KCl 5 mmol/L, CaCl_2_ 2.56 mmol/L, MgCl_2_ 1 mmol/L, NaHCO_3_ 10 mmol/L, HEPES 15 mmol/L, supplemented with 5.6 mmol/L glucose, 3 g of BSA/L, and equilibrated with a mixture of 95% O_2_/5% CO_2_ to obtain a pH = 7.4. This medium was then replaced with fresh buffer and the islets were incubated for 1 h with 2.8 and 16.7 mmol/L glucose. At the end of the incubation period, the supernatant was collected and insulin content measured by RIA.

### Western Blot

Insulin signaling experiments were conducted by western blot analysis. Briefly, mice were fasted overnight and received a single intraperitoneal injection of 100 µl (10^−6^ M) insulin [20] Tissues were harvested 5 min later. Gastrocnemius muscles and liver were homogenized in ice-cold buffer (Tris pH 7.5 100 mmol/L, sodium pyrophosphate 10 mmol/L, sodium fluoride 100 mmol/L, EDTA 10 mmol/L, sodium vanadate 10 mmol/L, PMSF 2 mmol/L and Triton X-100 1%) for 20 sec. Homogenates were softly agitated for 1 h at 4°C and subjected to centrifugation (14000 g for 40 min) at 4°C. Protein content in lysates was measured by the biuret dye method. For insulin receptor (IR) immunoprecipitation experiments, samples containing approximately 2 mg of protein were incubated with 10 µl of rabbit polyclonal anti-IR-β (SC-711, Santa Cruz, CA, USA) and incubated overnight at 4°C. Protein-antibody complexes were separated using protein A-sepharose (Amersham, Uppsala, Sweden), treated with Laemmli sample buffer containing dithiothreitol, then heated at 95°C for 5 min and loaded into 10% polyacrylamide gels. For experiments with total protein extracts, 70 µg were loaded into 10% gels. Following electrophoresis, proteins were transferred to nitrocellulose membranes and incubated overnight with blocking buffer (5% non-fat dried milk, Tris 10 mmol/L, NaCl 150 mmol/L, and Tween-20 0.05%). Membranes were then probed with primary antibodies against phosphotyrosine (SC-508), phospho Akt (Threonine^308^ and Serine^473^) (SC-16646-R, SC-7985-R, Santa Cruz, CA, USA), phospho ERK (SC-7383, Santa Cruz, CA, USA), and Phosphoinositide 3-kinase regulatory subunit (p85) (06-496, Upstate, Santa Cruz, NY, USA). Antibodies anti-Akt (SC-8312), anti-IRS-1 (SC-560; Santa Cruz, CA, USA) and anti-ERK (SC-135900, Santa Cruz, CA, USA) were used to normalize phosphorylated proteins. For total protein content, α-tubulin (SC-8035, Santa Cruz, CA, USA) was used as an internal control. Detection was performed using enhanced chemiluminescence (SuperSignal West Pico, Pierce, Rockford, IL, USA) after 2 h incubation with a horseradish peroxidase-conjugated secondary antibody (1∶10,000, Invitrogen, São Paulo, SP, BRA). Band intensities were quantified by optical densitometry (Scion Image, Frederick, MD, USA).

### Whole-Body Energy Homeostasis

Eleven to twelve BPA- and vehicle-treated mice were analyzed for energy expenditure, (respiratory quotient) RQ, and locomotor activity using a custom-made calorimetry system (LabMaster; TSE Systems). The instrument consists of a combination of highly sensitive feeding and drinking sensors for automated online measurement. The calorimetry system is an open-circuit system that determines O_2_ consumption, CO_2_ production, and RQ. A photobeam-based activity monitoring system detects and records every ambulatory movement, including rearing and climbing movements, in every cage. Lights were on from 7 am until 7 pm. Habituation to the metabolic cages consisted of 4 days of adaptation, after which animals were brought to normal cages during 3 days. For the analysis, animals were put back into metabolic cages for 48 h. The last 24 h, corresponding to the eight day of subcutaneous BPA administration (100 µg/kg/day), were used for data collection.

### Statistical analysis

Data is shown as mean ± SEM. Student's *t* test, one-way or two-way ANOVA were performed as appropriate with a level of significance p<0.05.

## Results

### BPA treatment impairs glucose homeostasis in adult mice and increase glucose-stimulated insulin secretion

**Figure 1:**
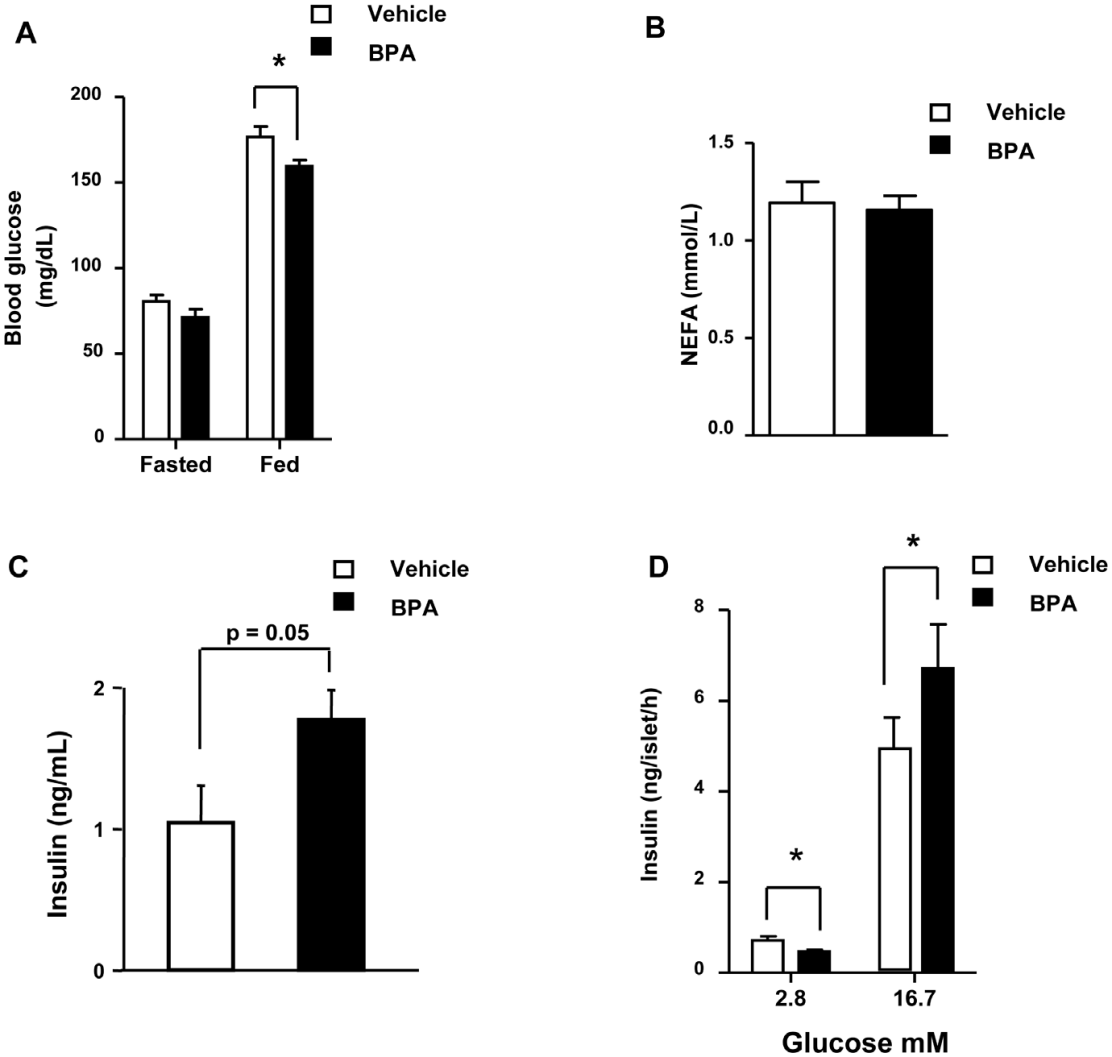
In vivo parameters and glucose stimulated insulin secretion in vehicle and BPA-treated mice for 8 days. (**A**) Blood glucose levels in fasted and fed state (n = 7–9), (**B**) Plasma non-esterified fatty acids (NEFA) (n = 7–9). (**C**) Plasma insulin (n = 6–8). (**D**) Glucose-induced insulin secretion in isolated islets from vehicle and BPA treated mice. Isolated islets were incubated with 2.8 or 16.7 mM glucose for 1 hour (n = 12). Statistical differences were determined by Student's t test *, p<0.05. Data are expressed as mean ±S.E.M.

To examine the effect of BPA on whole body metabolic parameters, we treated mice by subcutaneous injection of either corn oil vehicle or BPA at doses of 100 µg/kg/day for 8 consecutive days. BPA treatment for 8 days had no effect on body weight (Figure S1). Blood glucose levels were similar during fasting but decreased in the fed state in mice treated with BPA (Figure 1A). Lipid metabolism was likely unaltered in vehicle and BPA-treated mice since they presented similar levels of plasma non-esterified fatty acids (NEFA, Figure 1B). Plasma insulin in the fed state showed an increase in the BPA treated mice (p = 0.05) (Figure 1C).

We have previously shown that 4 days of treatment with 17β-estradiol or BPA at a dose of 100 µg/kg/day increase insulin content and release from pancreatic β-cells. Here, we confirm the hyperinsulinemic effect of BPA showing that eight days of treatment also led to an increase of glucose-stimulated insulin secretion (GSIS) in isolated islets (Figure 1D). Notably, basal insulin secretion was inhibited by BPA treatment (Figure 1D). These results strengthen the idea that BPA has a direct effect on pancreatic β-cells by increasing glucose-stimulated insulin secretion.

### BPA treatment alters whole body energy homeostasis in adult mice

**Figure 2:**
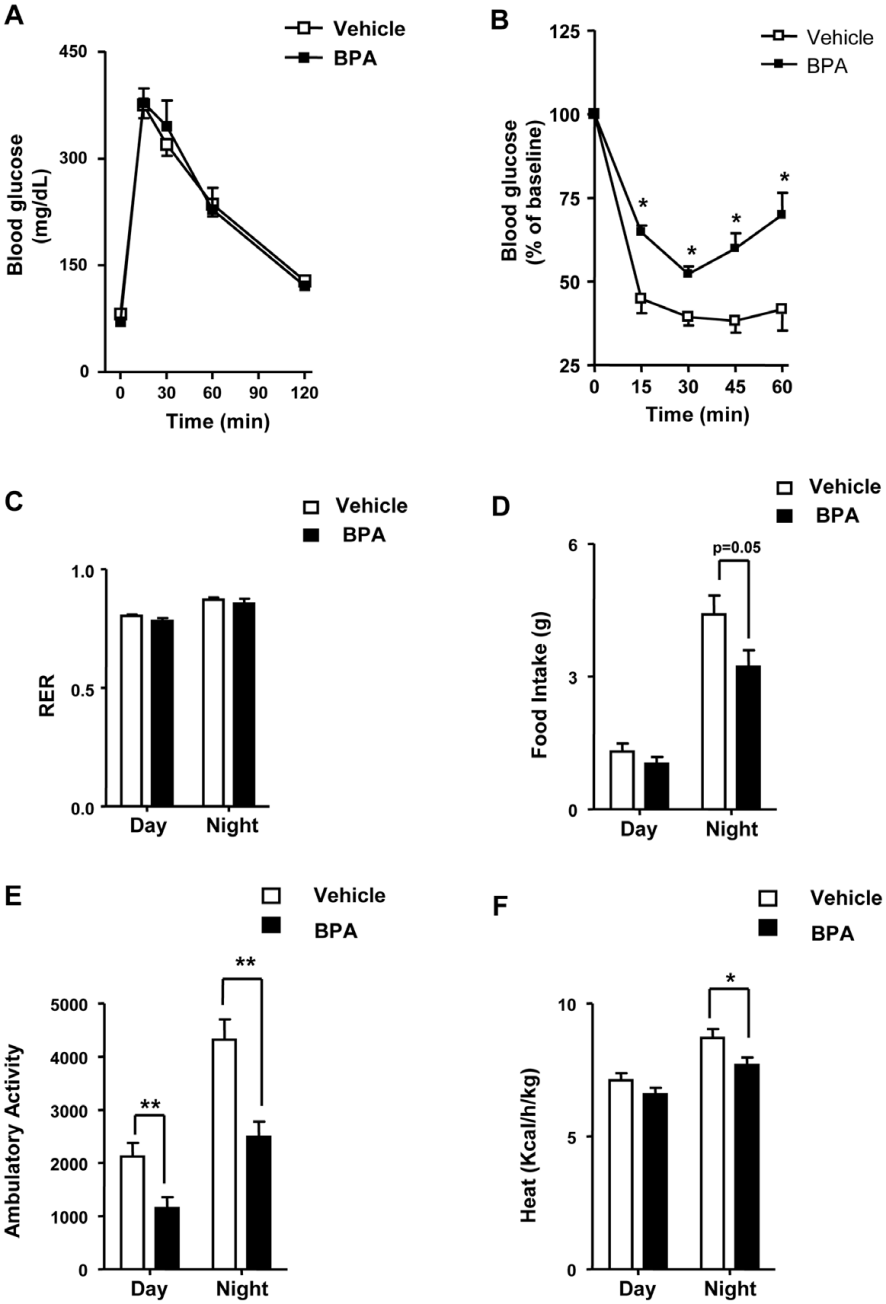
BPA treatment leads to insulin resistance and impairments in whole-body energy homeostasis. (**A**) Intraperitoneal glucose tolerant test in mice treated with vehicle or BPA. Plasma glucose concentration during the ipGTT (n = 7–8). (**B**) Intraperitoneal insulin tolerant test. Plasma glucose concentration during the ipITT (n = 7–8). (**C**) Respiratory exchange ratio (RER) assed over 24 h (n = 6). (**D**) Food Intake assed over 24 h (n = 6) (**E**) Ambulatory Activity assed over 24 h (n = 6). (**F**) Body temperature assed over 24 h (n = 6). Statistical differences were determined by Student's t test *, p<0.05; **, p<0.01. Data are expressed as mean ±S.E.M.

We next analyzed whether BPA treatment alters *in vivo* glucose and insulin tolerance (Figure 2). Whereas glucose clearance was similar between both groups upon glucose administration (Figure 2A), BPA-treated mice had reduced insulin sensitivity in comparison to control mice when insulin tolerance tests were performed (Figure 2B). To determine whether BPA treatment had an effect on metabolism, the respiratory exchange ratio (RER), was measured by indirect calorimetry over 24 h. This is a numeric index of carbohydrate and fat utilization based on a ratio of carbon dioxide produced to oxygen consumed. RER was unchanged between vehicle and BPA-treated mice (Figure 2C). The diurnal pattern of food intake was similar between vehicle and BPA-treated mice whereas the nocturnal pattern of food intake was decreased (p = 0.05) in BPA-treated mice (Figure 2D). Importantly, the diurnal and nocturnal 24-h rhythm of spontaneous locomotor behaviour was decreased in BPA-treated mice comparing to controls (Figure 2E). The decreased energy expenditure was also evident in BPA-treated mice (Figure 2F). These results demonstrate that BPA treatment leads to impairments in insulin action and alterations in whole-body energy homeostasis.

### BPA treatment alters insulin signaling in the skeletal muscle

**Figure 3:**
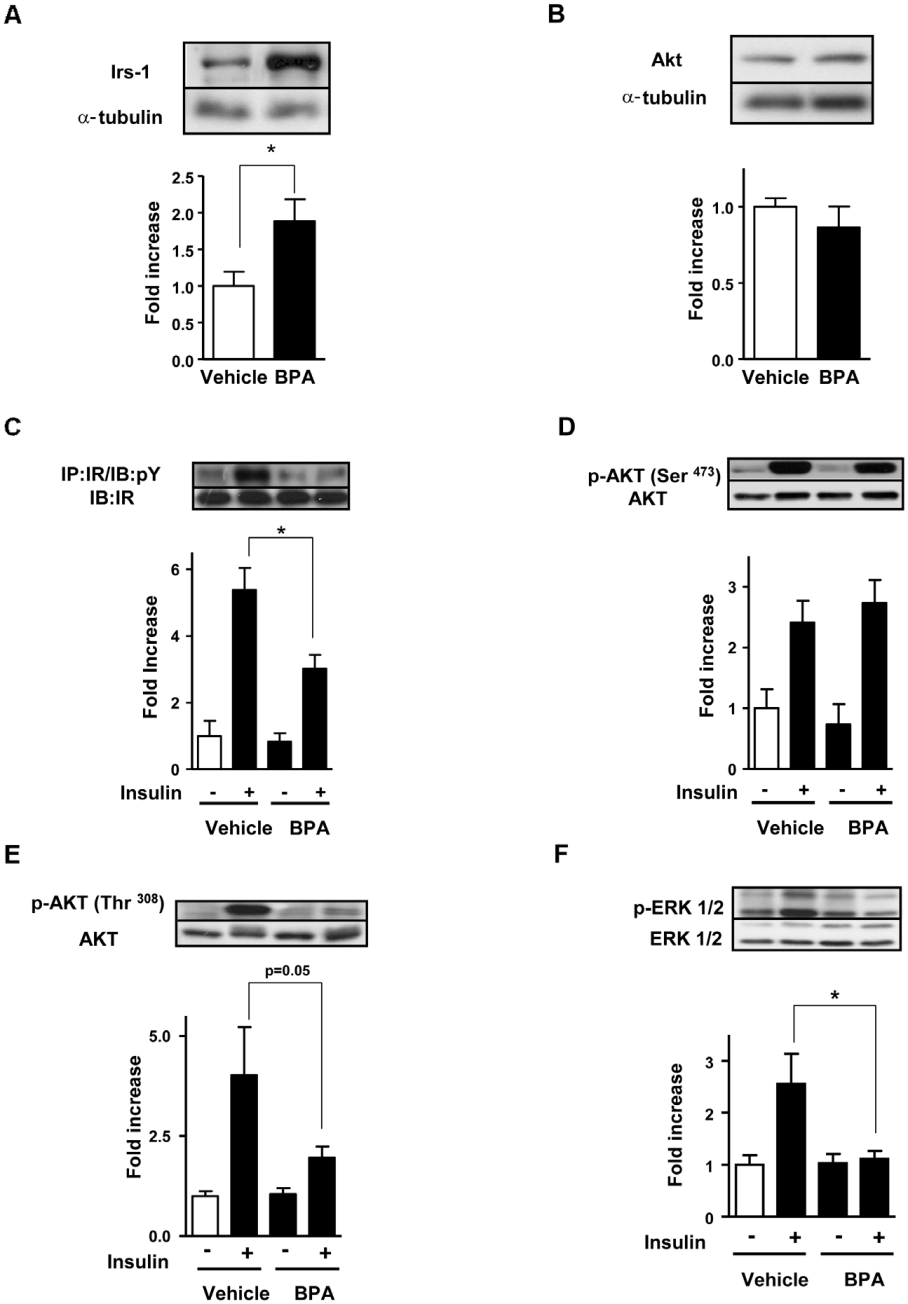
BPA treatment leads to increased IRS-1 protein in basal conditions and alters insulin signaling in insulin-stimulated conditions in skeletal muscle . (**A**) Total IRS-1 protein expression levels (n = 5). (**B**) Akt protein expression (n = 4). α-tubulin was used as an internal control. (**C**) IR tyrosine phosphorylation after insulin stimulation in vehicle and BPA treated animals (n = 5–7), (**D**) Akt phosphorylation (Ser^473^) (n = 5–7) and (**E**) Akt phosphorylation (Thr^308^) in the same conditions as panel C (n = 4–5) (**F**) ERK1/2 phosphorylation (n = 4–6). Statistical differences were determined by Student's t test *, p<0.05. Data are expressed as mean ±S.E.M.

Since treatment of BPA during adulthood leads to alterations in insulin action (Figure 2B), we next studied the insulin signaling pathways in the skeletal muscle of mice treated with BPA. In basal conditions, BPA treatment induced a 2-fold increase in the IRS-1 protein expression compared to vehicle (Figure 3A). The downstream pathways of the insulin receptor such as phosphoinositide 3-kinase (PI3K) subunit p85 (Figure S2A) and Akt protein expression were not affected by BPA-treatment in skeletal muscles (Figure 3B). Our results indicate that BPA treatment upregulates IRS-1 protein expression in skeletal muscle in basal conditions.

We next verified whether BPA treatment affects insulin signaling pathways in insulin stimulated conditions. Intraperitoneal insulin injection stimulated tyrosine phosphorylation in skeletal muscle from mice treated with vehicle within 5 minutes (Figure 3C), reflecting the physiological nature of the insulin stimulation. In contrast, insulin stimulation elicited a lower level of tyrosine phosphorylation of the insulin receptor β subunit in the skeletal muscle from BPA-treated mice (Figure 3C). Akt/PKB is a protein downstream mediator of PI3K that plays a important role in insulin stimulation of glucose transport. We assessed the most important phosphorylation sites of Akt protein involved in glucose uptake in skeletal muscle. Insulin-stimulated Akt phosphorylation in the Ser^473^ residue was similar in skeletal muscle from vehicle and BPA-treated mice (Figure 3D). Interestingly, Akt phosphorylation in the Thr^308^ was decreased (p = 0.05) in the skeletal muscle from BPA-treated mice (Figure 3E). These results indicate that short-treatment with BPA alters insulin signaling in skeletal muscles, which might contribute to the insulin resistance found in these mice.

The mitogen-activated protein kinase (MAPK) signaling pathway plays an important role in cell growth, proliferation, and differentiation in skeletal muscle and it is regulated by insulin as well. We next checked whether BPA treatment could affect the MAPK pathway in skeletal muscles. Figure 3F shows that basal ERK phosphorylation was similar between vehicle and BPA-treated mice. As expected, insulin increased the phosphorylation of ERK in the vehicle (Figure 3F). In contrast, insulin was not able to increase ERK phosphorylation in the muscles from BPA-treated mice (Figure 3F). These results show that BPA can impair insulin-stimulated MAPK signaling pathway. Thus, BPA downregulates multiple sites in the insulin signaling cascade in skeletal muscles.

### BPA treatment alters insulin signalling in the liver

**Figure 4:**
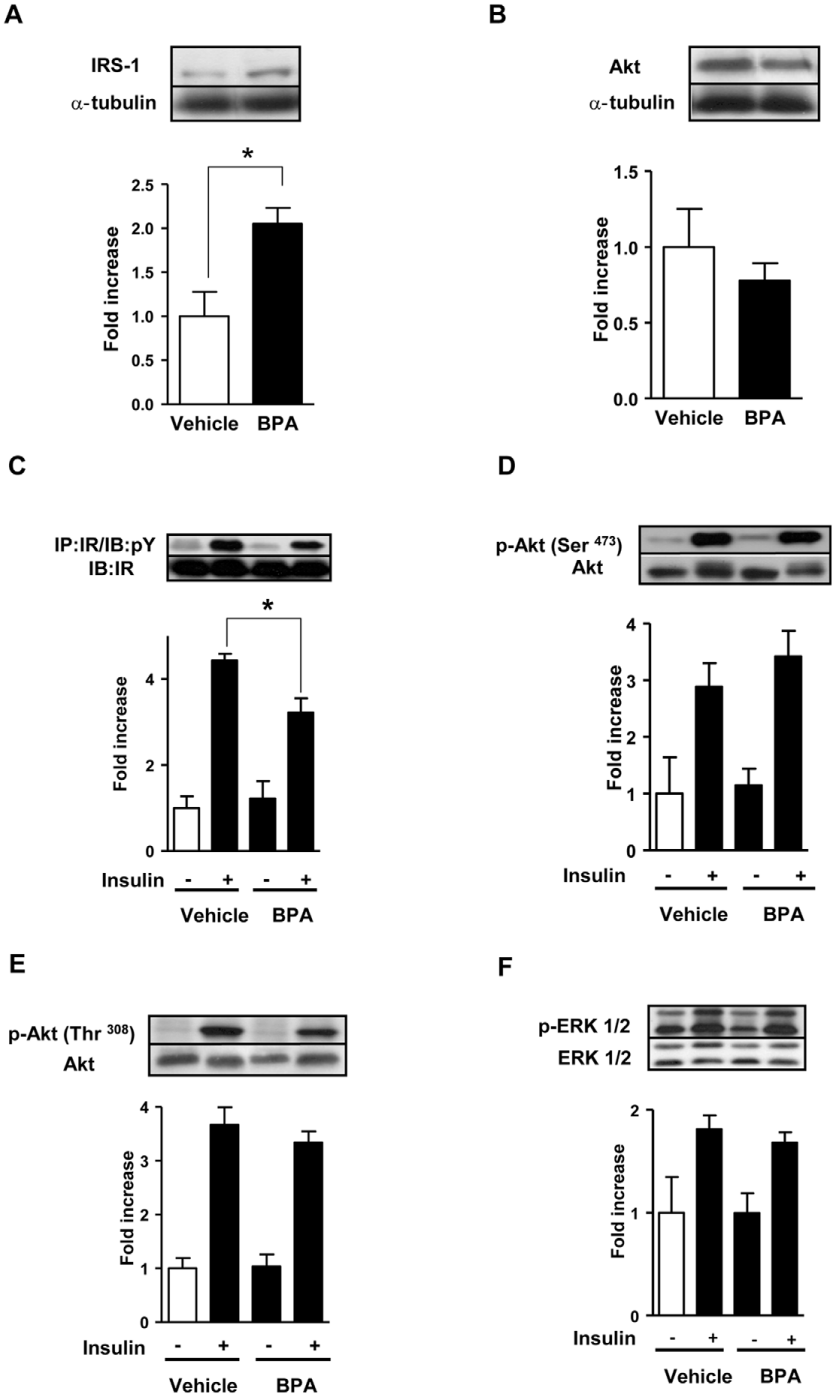
BPA treatment leads to increased IRS-1 protein in basal conditions and alters insulin signaling in insulin stimulated conditions in the liver. (**A**) Total IRS-1 protein expression (n = 4). (**B**) Akt protein expression (n = 5). α-tubulin was used as an internal control. (**C**) IR tyrosine phosphorylation (n = 5–7), (**D**) Akt phosphorylation (Ser^473^) (n = 5–7), (**E**) Akt phosphorylation (Thr^308^) (n = 5–7), (**F**) ERK1/2 phosphorylation (n = 4–7). Statistical differences were determined by Student's t test *, p<0.05. Data are expressed as mean ±S.E.M.

Insulin action in the liver is essential to shutdown the hepatic glucose production during postprandial states to control glucose homeostasis. As observed in the muscle, BPA treatment led to an upregulation of IRS-1 protein expression (Figure 4A), and did not affect PI3K subunit p85 (Figure S2B) and Akt protein expression (Figure 4B) in basal conditions in the liver. Impairments in the insulin signaling pathway were evident in liver from BPA-treated mice only at the level of the tyrosine phosphorylation of the insulin receptor β subunit, which was less phosphorylated upon insulin administration in BPA-treated mice in comparison to controls (Figure 4C). However, unlike in the skeletal muscle, downstream pathways in the insulin signaling remained unaffected by BPA treatment. Insulin-stimulated Akt phosphorylation in the Ser^473^ and Thr^308^ residue were similar in livers from vehicle and BPA-treated mice (Figure 4D and 4E). No differences were observed in the mitogen-activated protein kinase signaling in both basal and insulin stimulated conditions in livers from vehicle and BPA-treated mice (Figure 4F). We also investigated the effect of BPA treatment on pyruvate-induced gluconeogenesis by performing a pyruvate tolerance test. As shown in Figure S3, pyruvate tolerance was unaltered. These results show that BPA treatment can also downregulate part of the insulin signaling in the liver. Nonetheless, the insulin resistance observed in BPA-treated mice is mainly explained by a preferential targeting of BPA towards skeletal muscles.

## Discussion

In the present study we demonstrate that exposure to low doses of BPA during adulthood promotes adverse effects on glucose homeostasis and insulin action on peripheral tissues with the concomitant risk of developing type 2 diabetes.

Mice treated with BPA during 8 days presented no increase in weight and normal non-esterified fatty acids (NEFA) levels; however they presented insulin resistance and had a strong tendency to be hyperinsulinaemic in the fed state, together with decreased glucose levels. The hyperinsulinaemia in the fed state may be explained by an improved stimulus secretion coupling of β-cells, because islets isolated from BPA treated mice displayed a greater release of insulin in response to high glucose. The stimulatory action of BPA on islets may be due to an adaptation to the peripheral insulin resistance or to a direct action of BPA on β-cells or both. In any case, a direct action of BPA is very likely because it has been demonstrated in isolated islets from mice and rats that low doses of BPA potentiates GSIS [19], [21] as well as pancreatic insulin content [19]. Fluvestran blocks BPA action on GSIS pointing to an involvement of estrogen receptors [21]. Furthermore, BPA potentiation of GSIS in mouse and human involves ERβ [22]. The use of antiestrogens as well as estrogen receptor knockout mice indicates that the regulation of pancreatic insulin gene expression and content by BPA and Estradiol (E2) is dependent of the estrogen receptor ERα [19]. These actions of ERα involve a nonclassical mechanism, initiated outside the nucleus with ERK1/2 activation [19]. Downstream of ERK1/2, the activation of the transcription factor NeuroD1 regulates insulin gene transcription [23]. The estrogenic action of BPA through extranuclear ERα is initiated at concentrations as low as 1 nM, indicating that via this nonclassical estrogen activated pathway, BPA is as potent as the natural hormone E2 [19]. Estrogen receptors are essential molecules involved in glucose homeostasis and energy balance [24]–[26]. Using genetic rescue of nonclassical ERα signalling in ERα−/− mice, it has been demonstrated that energy homeostasis is greatly controlled by nonclassical ERα activated pathways [27]. Then, it is plausible that the effects of BPA on energy balance, glucose homeostasis and insulin sensitivity described in the present work are, at least in part, mediated by ERα in a nonclassical manner. Nevertheless, an action of BPA on ERβ, G protein-coupled receptor 30 (GPR30) or estrogen-related receptor γ (ERRγ) cannot be ruled out at the present moment [24], [28]–[30].

In the present work, BPA treatment led to changes in whole-body energy homeostasis. Mice treated with BPA showed the same levels of RER indicating that 8 days treatment may not be enough to change the use of substrate for energy. However, BPA treatment led to lower energy intake (measured by food consumption) and lower energy expenditure (measured by locomotor activity and heat production). Notably, body weight was unchanged. These changes could be explained by a direct effect of BPA on the central nervous system, because estrogen signaling modifies leptin and insulin responses and decreases food intake [31]. This may occur through the hypothalamus, since estrogen alters melanocortin cells rewiring and modulate energy balance [32], [33]. Similarly, other endocrine disruptors such as soy-derived isoflavones alter energy balance in association with changes in Agouti related Protein (*AgRP*) expression in the hypothalamus [34]. Nevertheless, a direct effect of BPA in other tissues cannot be excluded. We have recently shown that mice treated with BPA during pregnancy have increased leptin levels compared to controls at the end of pregnancy [16]. It is plausible that BPA could directly increase leptin levels through a direct action on adipocytes, since it can alter the release of adiponectin [35]. This could explain the decreased food intake found in our experiments with BPA-treated mice. In any case, we cannot rule out a CNS-related behavioral effect associated with BPA exposure; reduced food intake and locomotor activity may reflect lethargy. Overall, these results indicate that BPA treatment alters energy metabolism in mice.

Type 2 diabetes mellitus is characterized by insulin resistance, which results in lower levels of insulin-induced blood glucose uptake into target tissues. Here we showed that BPA disrupts insulin signaling in skeletal muscle and liver. In response to insulin, autophosphorylation of the insulin receptor (IR) is decreased in both skeletal muscle and liver by BPA treatment when compared to controls. Downstream Akt phosphorylation on Thr^308^ was, however, only decreased in skeletal muscles.

Several studies have provided evidence for defects in the insulin signaling in human skeletal muscle from obese and type 2 diabetes subjects using *in vitro* and *in vivo* approaches [36]. Our results in the skeletal muscle showed that BPA treatment led to decreased IR tyrosine phosphorylation, which was followed by reduced Akt phosphorylation on Thr^308^. However, comparable levels on insulin-induced Ser^473^ phosphorylation were detected in vehicle and BPA-treated mice. These observations are in agreement with similar studies in skeletal muscle from type 2 diabetic patients showing decreased insulin-induced Akt phosphorylation on Thr^308^ and similar levels of insulin-induced Ser^473^ phosphorylation [37]. Apart from its effects via Akt, insulin also activates the MAP kinase pathway to increase cell growth, proliferation, and differentiation [38], [39]. It has been shown that skeletal muscles from obese and type 2 diabetic subjects have normal insulin stimulation of the MAP kinase pathway [38]. Here we show that BPA-treatment impairs insulin-induced ERK phosphorylation in skeletal muscle. In adult animals, an increase in skeletal muscle mass occurs mainly as a result of an increase in the size rather than the number of muscle fibres and is generally thought to be regulated by the Akt/mTOR pathway [40]. Although the role that ERK1/2 activation may have in skeletal muscle it is still greatly unknown, this pathway is related with the increase in the expression of L-type amino acid transporter LAT2 after RSK1/2 phosphorylation [41]. In addition, ERK1/2 activation upregulates leptin receptor expression in C2C12 myotubes [42]. The decrease of insulin-induced ERK1/2 phosphorylation described here is an interesting finding that indicates a new potential deleterious effect of BPA on MAP kinase pathway, which is absent in obesity and type 2 diabetes but may be related to leptin resistance in skeletal muscle among other deleterious actions.

The effects of BPA treatment were less severe in the liver than in the skeletal muscle. In the liver, impairments of insulin signaling were only present at the level of IR phosphorylation. The normal insulin-stimulated Akt phosphorylation and ERK might be due to a compensation mechanism as a consequence of the upregulation of IRS-1. BPA treatment causes upregulation of IRS-1 protein expression in the absence of insulin in both tissues. This may counteract the diminished phosphorylation of the insulin receptor in the liver, avoiding insulin resistance. In skeletal muscle, although there is up regulation of IRS-1 at the basal level, this may not be powerful enough to counteract the BPA-decreased autophosphorylation of the insulin receptor, which is stronger in skeletal muscle than in the liver.

Skeletal muscle accounts for 75% of glucose regulation in the body and therefore it has a remarkable impact on blood glucose homeostasis. It is established that type 2 diabetes mellitus is characterized by insulin resistance, which results in lower levels of blood glucose uptake into target tissues. Consequently, blood glucose levels increase and more insulin is released producing hyperinsulinaemia, which is manifested early in type 2 diabetes. The effect of BPA on skeletal muscle may be direct on myocytes or a consequence of the higher insulin release from β-cells produced by BPA, or both. Several studies have demonstrated that the hypersecretion of insulin is a primary defect of type 2 diabetes and that insulin resistance develops secondarily to chronic hyperinsulinaemia [43]–[46]. Indeed, the persistance of chronic physiological euglycemic hyperinsulinaemia for 3–5 days can induce severe insulin resistance in healthy subjects with normal glucose tolerance [46]. Therefore it is plausible that the hyperstimulation of β-cells produced by BPA may result in producing insulin resistance in muscle and liver. This does not rule out a direct effect of BPA on peripheral tissues. No direct effect on insulin signalling has been yet described in neither the skeletal muscle nor the liver, but high doses of BPA in the micromolar range has been shown to affect leptin synthesis, to downregulate glucose transporters in adipocytes and enhance lipid accumulation in the liver [47]–[49].

Thus, the present work demonstrates that low doses of the environmental estrogen BPA in mice reduces overall energy metabolism and leads to impairments on insulin action in peripheral tissues, mainly in the skeletal muscle. These actions are triggered at BPA levels close to the tolerable daily intake (TDI) of 50 µg/kg/day set by the US-EPA and the EFSA. The TDI was calculated by decreasing 1000 fold the lowest observed adverse effect level (LOAEL) of 50 mg/kg/day. It could be argue that the route of administration in the present work is subcutaneous (s.c.) injection while the main route of human exposure is ingestion. We used s.c. injection because we need to know exactly the administered doses in order to properly perform mechanistic studies. However, it must be noted that we previously published that oral administration of BPA at exactly the same doses used in the present work produced insulin resistance, as manifested by alteration of insulin tolerance test [15]. Moreover, a previous study by Prins et al [50] stated that “…. despite differences in BPA metabolism, clearance and excretion mechanisms that diverge between rodents and humans and despite differences in BPA pharmacokinetics in route of exposure, the s.c. delivery of BPA employed by our laboratory provides an internal dose and tissue bioavailability that models internal human levels”. Therefore the results presented in this work may be relevant to humans.

During the recent years results have accumulated that indicate BPA effects at lower exposures [6]. New pharmacokinetics experiments performed in Rhesus monkeys, assuming a similar metabolism to humans, point towards a BPA exposure as high as 500 µg/kg/day. Moreover, epidemiological studies clearly associate BPA levels in urine with risk of type 2 diabetes [7], [51] and remarkably, with insulin resistance in individuals with normal body mass index [8]. Taken together with the fact that BPA exposure is widespread, bisphenol-A can thus be considered as a risk factor for type-2 diabetes.

## Supporting Information

Figure S1 **Body weight of mice treated with vehicle or BPA for 8 days (n = 8).**
(TIF)

Figure S2 **Total PI3K (p85) protein expression.** A) Total PI3K regulatory subunit (p85) protein expression (n = 5). B) Total PI3K regulatory subunit (p85) protein expression (n = 4).
(TIF)

Figure S3 **Pyruvate Tolerance Test.** Mice received an intraperitoneal injection of sodium pyruvate (2 g/Kg body weight) diluted in saline after a 16 hour fast. Blood glucose was then determined at the indicated time points (n = 8–9).
(TIF)
