# Supplementary figures and images for: Short-Term Treatment with Bisphenol-A Leads to Metabolic Abnormalities in Adult Male Mice
Source: PLoS One. 2012 Mar 28;7(3):e33814. doi: 10.1371/journal.pone.0033814 (PMC3314682; doi:10.1371/journal.pone.0033814)

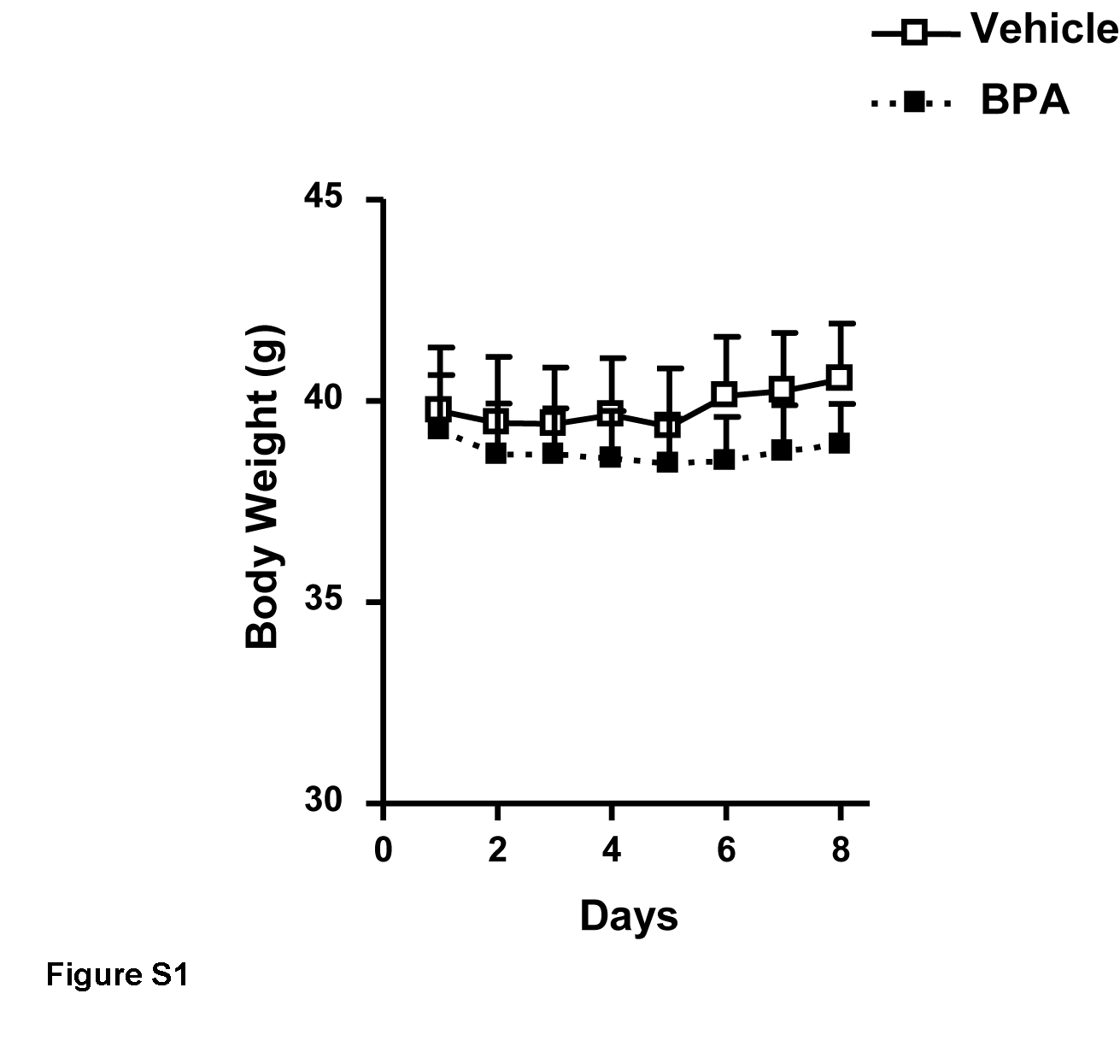

Supplement: Figure S1 — Body weight of mice treated with vehicle or BPA for 8 days (n = 8). (TIF) [file pone.0033814.s001.tif]

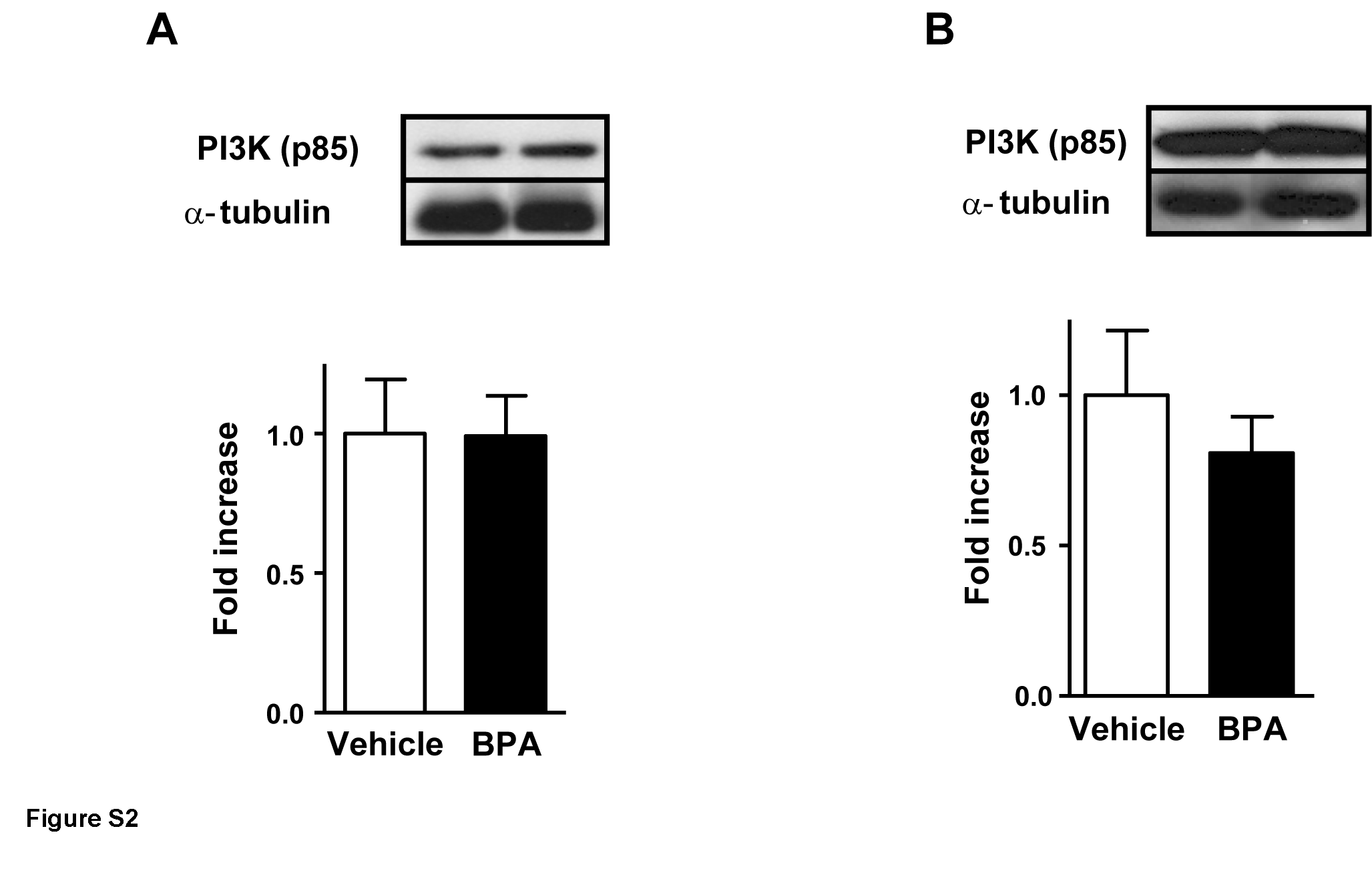

Supplement: Figure S2 — Total PI3K (p85) protein expression. A) Total PI3K regulatory subunit (p85) protein expression (n = 5). B) Total PI3K regulatory subunit (p85) protein expression (n = 4). (TIF) [file pone.0033814.s002.tif]

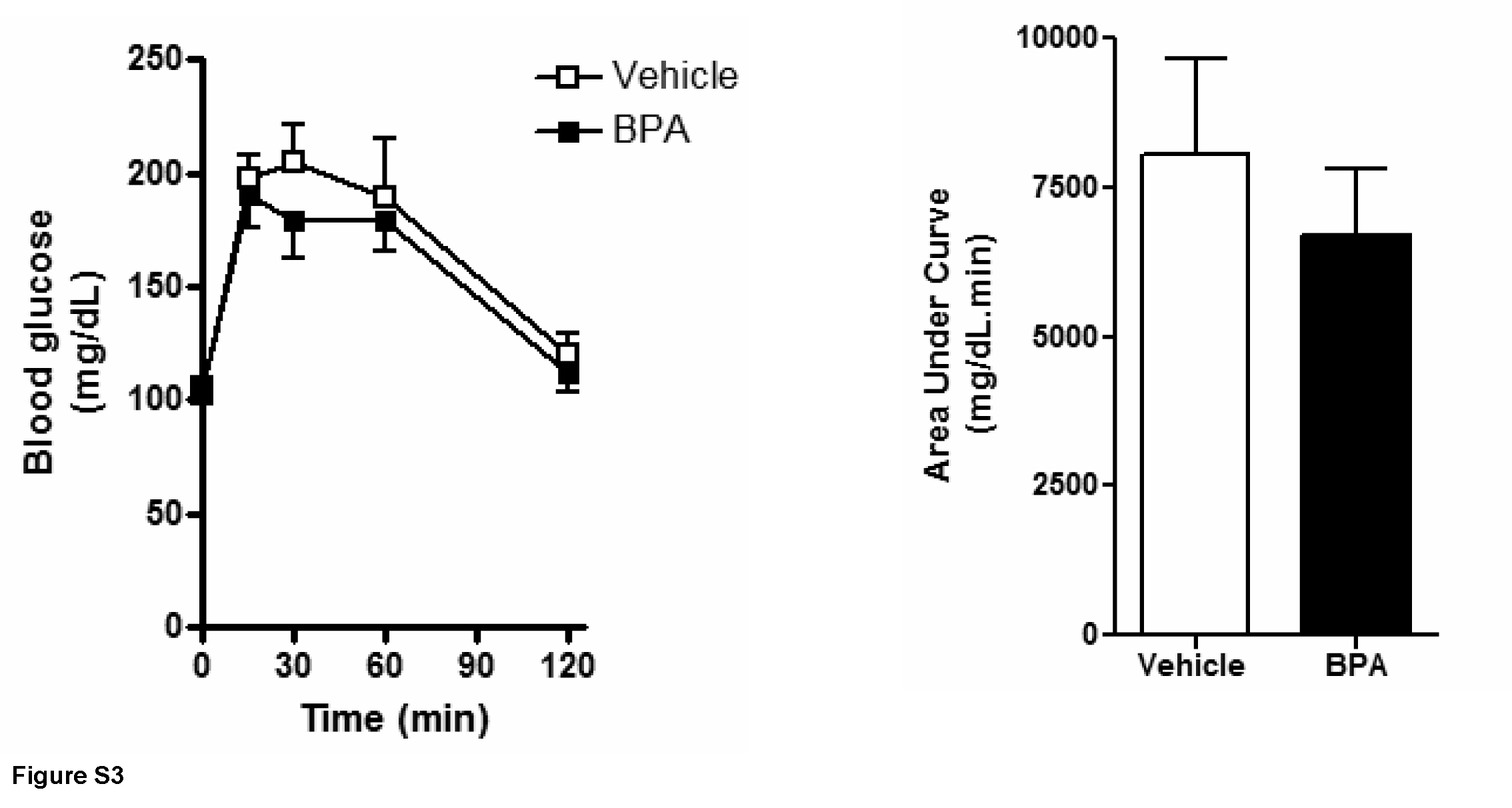

Supplement: Figure S3 — Pyruvate Tolerance Test. Mice received an intraperitoneal injection of sodium pyruvate (2 g/Kg body weight) diluted in saline after a 16 hour fast. Blood glucose was then determined at the indicated time points (n = 8–9). (TIF) [file pone.0033814.s003.tif]
